# Forensic investigation of falsified antimalarials using isotope ratio mass spectrometry: a pilot investigation

**DOI:** 10.1038/s41598-024-54168-9

**Published:** 2024-02-18

**Authors:** Paul N. Newton, Lesley A. Chesson, Mayfong Mayxay, Arjen Dondorp, Patricia Tabernero, John D. Howa, Thure E. Cerling

**Affiliations:** 1 Lao-Oxford-Mahosot Hospital-Wellcome Research Unit (LOMWRU), Microbiology Laboratory, Mahosot Hospital, Vientiane, Lao PDR; 2 Centre for Tropical Medicine and Global Health, Nuffield Department of Medicine, University of Oxford, Oxford, UK; 3 Infectious Diseases Data Observatory, Nuffield Department of Medicine, University of Oxford, Oxford, UK; 4 IsoForensics, Inc, Salt Lake City, UT USA; 5 Institute of Research and Education Development (IRED), University of Health Sciences, Ministry of Health, Vientiane, Lao PDR; 6 Mahidol Oxford Research Unit (MORU), Faculty of Tropical Medicine, Mahidol University, Bangkok, Thailand; 7 Public Health Unit, Faculty of Medicine, Universidad de Alcalá, Alcalá de Henares, Spain; 8 Department of Geology and Geophysics and Department of Biology, University of Utah, Salt Lake City, UT USA

**Keywords:** Stable isotope, Isotope ratio mass spectrometry (IRMS), Forensic science, Drugs, Isotopic profiling, Malaria, Falsified medicine, Substandard medicine, Medicine quality, Clinical pharmacology, Malaria

## Abstract

We explored whether isotope ratio mass spectrometry (IRMS) is useful to investigate the origin of falsified antimalarials. Forty-four falsified and genuine antimalarial samples (artesunate, artemether-lumefantrine, dihydroartemisinin-piperaquine and sulphamethopyrazine-pyrimethamine) were analyzed in bulk for carbon (C), nitrogen (N), and oxygen (O) element concentrations and stable isotope ratios. The insoluble fraction (“starch”) was extracted from 26 samples and analyzed. Samples of known geographical origin maize, a common source of excipient starch, were used to produce a comparison dataset to predict starch source. In both an initial (*n* = 18) and a follow-on set of samples that contained/claimed to contain artesunate/artemether (*n* = 26), falsified antimalarials had a range of C concentrations less than genuine comparator antimalarials and δ^13^C values higher than genuine comparators. The δ^13^C values of falsified antimalarials suggested that C_4_ plant-based organic material (e.g., starch derived from maize) had been included. Using the known-origin maize samples, predictions for growth water δ^18^O values for the extracted “starch” ranged from − 6.10 to − 1.62‰. These findings suggest that IRMS may be a useful tool for profiling falsified antimalarials. We found that C_4_ ingredients were exclusively used in falsified antimalarials versus genuine antimalarials, and that it may be possible to predict potential growth water δ^18^O values for the starch present in falsified antimalarials.

## Introduction

A key tenet of malaria control lies in antimalarial treatments being accessible, affordable, and effective^1^. Poor quality antimalarials are thus a cause for serious concern. Falsified medical products are defined by the World Health Organization (WHO) as those “that deliberately/fraudulently misrepresent their identity, composition or source,” in contrast to substandard or “out of specification” medicines that “are authorized medical products that fail to meet either their quality standards or their specifications, or both”^2^.

Falsified medicines may contain no or incorrect active pharmaceutical ingredients (APIs), or incorrect API amounts, and impurities or poor bioavailability. Incorrect unstated excipients may also be damaging. Falsified medicines harm patients, weaken health systems, damage economies and, for antimicrobials, endanger antimicrobial resistance. WHO estimated that globally ~ 10% of medicines in low- and middle-income countries are substandard or falsified^2^. There have been numerous examples of falsified antimalarials, particularly in Southeast Asia and Sub-Saharan Africa, which must have had a major negative impact on malaria morbidity and mortality^2–5^. Their sources remain poorly understood and to provide actionable evidence innovative methods are needed to identify their origins and trade routes^6^.

There has been minimal research published on innovation of forensic techniques to trace where falsified medicines were manufactured, or their ingredients sourced. Pollen and calcium carbonate analyses were important in providing evidence that falsified oral antimalarial artesunate—in a large epidemic that afflicted mainland Southeast Asia in the late 1990s and early 2000s—was from southern China ^6^. More recently, environmental DNA has been shown to offer promise for providing signatures, specific for time and place, for the ingredients and manufacturing sites of falsified medicines^7^. There has been much more research and significant recent innovations in the forensic investigation of the illegal wildlife trade to provide evidence, for instance, on the habitat of elephant victims of the ivory trade. This uses stable isotope ratio mass spectrometry (IRMS) to measure small differences in amounts of isotopes in materials that are characteristic of geographic origin^8–11^.

Most elements in the periodic table have multiple isotopic forms, distinguished by different numbers of neutrons. Isotopes can be considered Nature’s recorders, useful for reconstructing biological, chemical, and ecological processes^8^. For example, carbon and nitrogen isotope delta (δ) values can be used to trace the fixation and movement of carbohydrates and protein, while oxygen isotope δ values can provide geolocation information as they are related to the systematic global variations in environmental water. Natural and artificial transfers lead to changes in relative amounts of stable isotopes within materials, in a phenomenon called isotopic fractionation^12–23^.

Differences in the carbon isotopic composition of biological material can be related to differences in the photosynthetic pathways of plants. The C_3_ pathway, used by temperate grasses, trees, and crops such as rice and wheat, typically results in relatively low δ^13^C values for plant tissues (e.g., − 35 to − 20‰) while the C_4_ pathway, used by tropical grasses and crops such as maize and sugar cane, typically results in higher values (e.g., − 14 to − 10‰)^14^. Due to geochemical processes, fossil fuels generally have δ^13^C values lower than modern C_3_ plants, ranging between − 60 to − 20‰, while most marine carbonates (e.g., limestone and dolomite) have δ^13^C values higher than C_4_ plants, ranging between approximately − 5 and + 2‰^15^.

Differences in δ^15^N values within the environment are influenced by chemical changes within the nitrogen cycle. Common inputs to the terrestrial part of the cycle include atmospheric nitrogen deposition (soils) and nitrogen fixation (plants) while outputs include gaseous losses and hydrologic leaching. The isotopic fractionation factors associated with these processes are often dependent upon nitrogen quantity and the conditions (e.g., temperature, aridity, enzyme properties, etc.) under which nitrogen is cycled^16–18^.

The oxygen isotopic composition of terrestrial surface water varies with geographic location in a predictable manner^19–21^. Precipitation causes isotopic fractionation as water molecules move from oceans onto land surfaces^22^. The amount of fractionation varies with distance from the ocean, elevation, and temperature. Surface water in warmer climates typically has higher δ^18^O values than water found in colder, higher latitude locations. The local water signals resulting from this predictable isotopic fractionation are transferred to plants and animals and recorded in their tissues e.g., plant carbohydrate through photosynthesis. Once recovered, this signal can provide geolocation information^23^. Plants and animals with lower δ^18^O values more likely originated from colder climates, higher elevations, and/or regions more inland than plants or animals with higher δ^18^O values.

IRMS techniques have been used for the characterization of both illicit drugs and medications. For illicit drugs, isotopic profiling has been useful for collecting source intelligence, elucidating production processes, and making sample-to-sample comparisons^24–39^. For medications, isotopic profiling can aid in the authentication of genuine pharmaceuticals and, conversely, the detection of falsified medicines^40–49^. We investigated the δ^13^C and δ^18^O values of calcite in genuine and falsified artesunate antimalarials, suggesting that the calcite was of high temperature intrusive origin, probably in southern China^6^.

We therefore conducted a further pilot study to assess whether stable isotope analysis of falsified antimalarial medicines could provide evidence of their source. We used both bulk and component-specific approaches for isotopic profiling. In an initial sample set, 18 antimalarial samples, stated to contain artesunate, artemether-lumefantrine, dihydroartemisinin-piperaquine and sulphamethopyrazine-pyrimethamine, were analyzed without any prior purification (i.e., bulk) for carbon (C), nitrogen (N), and oxygen (O) element concentrations and stable isotope ratios. In a follow-on set, 26 antimalarial samples, stated as including artesunate or artemether-lumefantrine, were first analyzed as bulk material before an aliquot of each was extracted using a series of different solvents to collect the insoluble fraction (“starch”); that fraction was also analyzed for element concentrations and stable isotope ratios. Starches extracted from samples of known origin maize (*Zea mays*), a common excipient, were used to produce a comparison dataset to determine whether predictions could be made on the potential source of starch found in falsified antimalarials.

## Methods

### Samples

The medicine samples were collected as part of studies in the Lao PDR, Cambodia, Angola, Cameroon, China, and Myanmar^5,6,50–52^. All genuine samples were stated as containing starch and all but two falsified samples (1/29 and 2/12056) were recorded as containing starch (Supplementary Material-Tables 1 and 2). Information provided with samples included unique identification code, brand/stated manufacturer, and quality classification (genuine vs. falsified, based on previous HPLC, LC–MS, and packaging analyses^6^).

**Table 1:** Samples (*n* = 18) included in the initial study of antimalarials, with measured element concentrations and stable isotope ratios analyzed in bulk.

ID code	API	Brand/Stated manufacturer	Classification	wt%C	wt%N	wt%O	*δ*^13^C (‰)		*δ*^18^O (‰)		Mean	SD
							Mean	SD	Mean	SD		
China 07/04	Artesunate	Artesunate/Guilin	Genuine	43	–	43	− 19.6	0.09	–	–	28.8	0.27
China 07/10	Artesunate	Artesunate/Guilin	Genuine	43	–	45	− 19.5	0.23	–	–	29.3	0.30
G 15/2	Artesunate	Artesunate/Guilin	Genuine	42	–	43	− 20.6	0.17	–	–	28.8	0.02
1/29	Artesunate	Artesunate/Guilin	Falsified	13	–	30	− 11.8	0.20	–	–	10.3	0.09
1/13	Artesunate	Artesunate/Guilin	Falsified	24	–	34	− 11.1	0.06	–	–	26.0	0.58
2/12056	Artesunate	Artesunate/Guilin	Falsified	30	–	38	− 10.4	0.01	–	–	23.5	0.51
5/17	Artesunate	Artesunate/Guilin	Falsified	36	–	48	− 16.3	0.12	–	–	30.9	0.04
2/12012	Artesunate	Artesunate/Guilin	Falsified	40	–	41	− 16.1	0.04	–	–	28.5	0.32
5/22	Artesunate	Artesunate/Traphaco	Genuine	41	–	50	− 25.5	0.07	–	–	25.1	0.09
G-NOV-65	Artemether-lumefantrine	Coartem/Novartis	Genuine	54	1.2	21	− 27.6	0.01	2.3	0.19	26.7	0.22
Ao-2012–2	Artemether-lumefantrine	Coartem/Novartis	Falsified	12	–	33	− 12.4	0.01	–	–	11.7	1.02
NOV 10/43	Artemether-lumefantrine	Riamet/Novartis	Genuine	55	1.2	25	− 27.6	0.01	2.3	0.15	26.7	0.35
G-26/4	Dihydroartemisinin-piperaquine	Duo-Cotecxin/HolleyPharm	Genuine	36	5.3	31	− 25.2	0.16	2.3	0.04	15.7	0.01
China 07/18	Dihydroartemisinin-piperaquine	Duo-Cotecxin/HolleyPharm	Falsified	37	–	46	− 11.7	0.05	–	–	26.8	0.04
China 07/21	Dihydroartemisinin-piperaquine	Duo-Cotecxin/HolleyPharm	Falsified	36	–	48	− 11.5	0.08	–	–	27.0	0.02
UG 09/01–2	Sulphamethopyrazine-pyrimethamine	Metakelfin/Pfizer	Genuine	45	15.8	23	− 27.7	0.02	3.4	0.03	16.8	0.13
TAN 09/02–4	Sulphamethopyrazine-pyrimethamine	Metakelfin/Pfizer	Genuine	45	15.6	23	− 27.8	0.04	2.4	0.05	17.7	0.09
TAN 09/01–1	Sulphamethopyrazine-pyrimethamine	Metakelfin/Pfizer	Falsified	21	–	44	− 19.3	0.22	–	–	18.2	0.38

*SD* standard deviation, *API* active pharmaceutical ingredient, *Guilin* Guilin Pharmaceutical Co., Ltd as stated manufacturer.

**Table 2:** Samples (*n* = 26) included in the follow-on study of artesunate/artemether antimalarials with bulk C, N, and O element concentrations and stable isotope ratios.

ID code	API	Brand/Stated manufacturer	Classification	Bulk tablets						“Starch” fraction			
				wt%C	wt% N	wt% O	*δ*^13^C (‰)	*δ*^15^N (‰)	*δ*^18^O (‰)	wt% C	wt% O	*δ*^13^C (‰)	*δ*^18^O (‰)
G020	Artesunate	Artesan Pharma GmbH & Co.KG	Genuine	47	15.5	20	− 26.1	3.8	20.6	38	45	− 18.1	29.7
G021	Artesunate	Artesan Pharma GmbH & Co.KG	Genuine	43	12.2	30	− 25.9	3.8	26.2	41	46	− 23.8	31.9
1/1	Artesunate	Guilin Pharmaceutical Co., Ltd	Genuine	43	–	46	− 19.4	–	27.4	40	48	− 17.5	26.4
G053	Artesunate	Guilin Pharmaceutical Co., Ltd	Genuine	41	–	44	− 19.4	–	26.6	40	40	− 18.3	19.8
G050	Artesunate	Guilin Pharmaceutical Co., Ltd	Genuine	41	–	43	− 19.5	–	26.8	41	46	− 17.9	25.1
G052	Artesunate	Guilin Pharmaceutical Co., Ltd	Genuine	41	–	46	− 19.3	–	26.8	40	49	− 17.6	26.0
G015	Artesunate	Guilin Pharmaceutical Co., Ltd	Genuine	41	–	45	− 20.2	–	27.6	40	49	− 17.9	26.0
G215	Artesunate-amodiaquine	MAPHAR-MAROC	Genuine	44	4.5	25	− 25.6	− 2.8	19.9	30	43	− 21.2	26.4
G225	Artesunate-amodiaquine	MAPHAR-MAROC	Genuine	44	4.5	25	− 25.5	− 2.6	19.5	32	32	− 21.9	26.2
G226	Artesunate-Amodiaquine	MAPHAR-MAROC	Genuine	44	4.6	24	− 25.9	− 2.7	19.1	32	34	− 21.7	25.9
G227	Artesunate-amodiaquine	MAPHAR-MAROC	Genuine	44	4.3	26	− 25.7	− 3.1	21.1	31	43	− 21.4	26.6
Lao 07/25	Artesunate	Mediplantex	Genuine	43	–	45	− 27.7	–	27.4	40	47	− 26.7	28.3
G049	Artesunate	Mekophar Chemical Pharmaceutical Joint-Stock Co., Vietnam	Genuine	41	–	48	− 22.5	–	25.1	37	46	− 18.8	29.0
G054	Artesunate	Mekophar Chemical Pharmaceutical Joint-Stock Co., Vietnam	genuine	41	–	49	− 23.1	–	25.7	39	47	− 18.6	28.9
Lao 07/24	artesunate	Mekophar Chemical Pharmaceutical Joint-Stock Co., Vietnam	Genuine	39	–	46	− 22.7	–	24.3	39	48	− 19.5	27.9
Lao 05/22	Artesunate	Traphaco	Genuine	42	–	48	− 25.6	–	23.6	37	44	− 25.8	31.1
G069	Artemether	Cipla Ltd. India	Genuine	49	–	28	− 27.2	–	28.0	39	42	− 25.8	29.8
13011/1	Artesunate	Guilin Pharmaceutical Co., Ltd	Falsified	30*	–	46	− 10.8	–	23.9	32	39	− 11.3	23.7
13011/2	Artesunate	Guilin Pharmaceutical Co., Ltd	Falsified	30*	–	39	− 10.8	–	23.8	31	34	− 11.3	24.4
12071	Artesunate	Guilin Pharmaceutical Co., Ltd	Falsified	30*	–	38	− 10.7	–	23.7	31	39	− 11.4	23.1
12063	Artesunate	Guilin Pharmaceutical Co., Ltd	Falsified	29*	–	41	− 10.7	–	23.7	31	38	− 11.4	23.2
CAM S5 1/08	Artesunate	Mekophar Chemical Pharmaceutical Joint-Stock Co., Vietnam	Falsified	38	1.1	48	− 12.6	− 0.1	24.9	40	51	− 11.8	28.8
CAM S5 2/08	Artesunate	Mekophar Chemical Pharmaceutical Joint-Stock Co., Vietnam	Falsified	37	1.0	47	− 12.6	− 0.2	24.7	40	48	− 11.7	28.8
CAM S5 3/08	Artesunate	Mekophar Chemical Pharmaceutical Joint-Stock Co., Vietnam	Falsified	39	1.1	48	− 12.5	− 0.2	24.9	40	48	− 11.7	28.7
CAM S5 4/08	Artesunate	Mekophar Chemical Pharmaceutical Joint-Stock Co., Vietnam	Falsified	39	–	46	− 12.9	–	24.7	40	48	− 11.8	28.6
CAM S5 5/08	Artesunate	Mekophar Chemical Pharmaceutical Joint-Stock Co., Vietnam	Falsified	39	–	48	− 12.6	–	24.7	36	49	− 11.8	28.7

*Samples have element concentrations much smaller or larger than the ranges seen in pure starch reference materials.

*API* active pharmaceutical ingredient.

### Initial set of antimalarials (n = 18)

At least one tablet of each sample was available for analysis; in some cases, two tablets were available. The active pharmaceutical ingredients (APIs) included were artesunate (4 genuine and 5 falsified), artemether-lumefantrine (2 genuine and 1 falsified), dihydroartemisinin-piperaquine (1 genuine and 2 falsified), and sulphamethopyrazine-pyrimethamine (2 genuine and 1 falsified) (Table 1). The Supplementary Material includes further details on excipient content as determined by ATR-FTIR.

Tablets were ground to a fine powder using a ceramic mortar and pestle. In cases where two tablets were provided, only one tablet was ground. In cases where one tablet was provided, the single tablet was cut in half using a clean razor blade and only half the tablet was ground. One sample was largely powdered upon receipt and all material was subsequently ground. Ground material was stored in capped 1-dram glass vials at room temperature. Samples were analyzed in duplicate and means presented.

### Follow-on set of artesunate/artemether antimalarials (n = 26)

Between one and five tablets of each sample were available for analysis (Table 2). All tablets available per sample were ground to a fine powder using a ceramic mortar and pestle. Ground material was stored in capped 1-dram glass vials at room temperature. To isolate the “starch” fraction, a 250 mg aliquot of each powdered sample was extracted using 2 mL deionized water, then 2 mL 95% ethanol, 2 mL acetone, and finally 2 mL hexanes (Fisher Scientific). In each extraction, powder and solvent were mixed well and then centrifuged (1600 g for 3 min), the supernatant carefully decanted, and the insoluble fraction was extracted once more with the same solvent for a total of two extractions per solvent. The insoluble fraction remaining after all solvent extractions was air dried at room temperature. The dried starch fractions were stored in capped 1-dram glass vials at room temperature.

### Survey of plant starches (n = 21)

Nine fresh ears of maize were collected in the continental USA and 12 fresh ears of maize were collected from Asia and Africa, from retail outlets (Supplementary Material-Table 3). Maize collection complied with available institutional, national, and international guidelines and legislation. To extract starch, kernels were steeped in warm water for 24 h and then grated using a kitchen grater and the grated kernels covered with deionized water and soaked and agitated for five minutes. The liquid was left undisturbed until starch precipitated, and the supernatant was then carefully decanted. Solids were filtered using a paper coffee filter from the liquid and discarded. The starch was resuspended in 1–2 ml of deionized water and then centrifuged at 1600 g for 3 min to pellet starch. The supernatant was removed without disturbing the pellet. The starch remaining after decanting was extracted with the same series of solvents used to extract starch from antimalarials.

**Table 3:** Location of collected maize samples and estimated mean annual precipitation (MAP) *δ*^18^O values and average growing season precipitation (GSP) *δ*^18^O value for each collection location.

ID	Source	Latitude	Longitude	Elevation (m)	wt% O	starch *δ*^18^O (‰)	MAP *δ*^18^O (‰)	GSP *δ*^18^O (‰)
7	Michigan, USA	41.83	− 86.36	221	45	25.9	− 7.9	− 3.4
3	New Jersey, USA	39.78	− 74.86	38	40	25.6	− 7.9	− 3.8
5	Oklahoma, USA	35.96	− 97.23	267	39	27.7	− 6.8	− 3.1
39	Oregon, USA	43.61	− 123.26	61	37	27.3	− 11.1	− 4.3
33	Pennsylvania, USA	40.61	− 77.73	244	37	23.6	− 8.7	− 5.2
2	South Carolina, USA	32.79	− 80.11	0	44	28.0	− 4.9	− 3.7
41	Texas, USA	33.18	− 97.29	227	42	22.4	− 5.6	− 2.6
17	Utah, USA	40.89	− 111.88	1335	49	29.4*	− 13.2*	− 8.9*
35	Virginia, USA	38.77	− 77.73	157	40	26.3	− 7.7	− 3.9
18	Bangladesh	24.76	91.59	17	45	29.9	− 4.3	− 2.0
21	Cambodia	13.09	103.17	14	48	25.6	− 6.1	− 6.0
20	Lao PDR	20.97	101.41	555	44	20.2	− 7.3	− 7.3
24	Myanmar	16.56	98.57	224	47	23.2	− 6.1	− 4.9
26	Myanmar	16.32	98.66	264	47	26.5	− 6.2	− 5.1
27	Myanmar	16.34	98.66	254	45	25.5	− 6.2	− 5.0
19	Thailand	17.72	98.92	525	43	19.4	− 6.8	− 5.1
25	Thailand	16.83	98.54	183	47	24.9	− 6.0	− 4.3
28	Thailand	18.84	98.57	1030	44	24.5	− 7.8	− 6.2
10	DRC	− 4.39	15.97	509	27	20.2	− 4.4	− 2.7
22	Kenya	− 3.34	39.77	149	46	29.9	− 2.0	− 2.1
23	Kenya	1.01	34.96	1823	44	34.5	− 4.2	− 2.7

**Figure 1:**
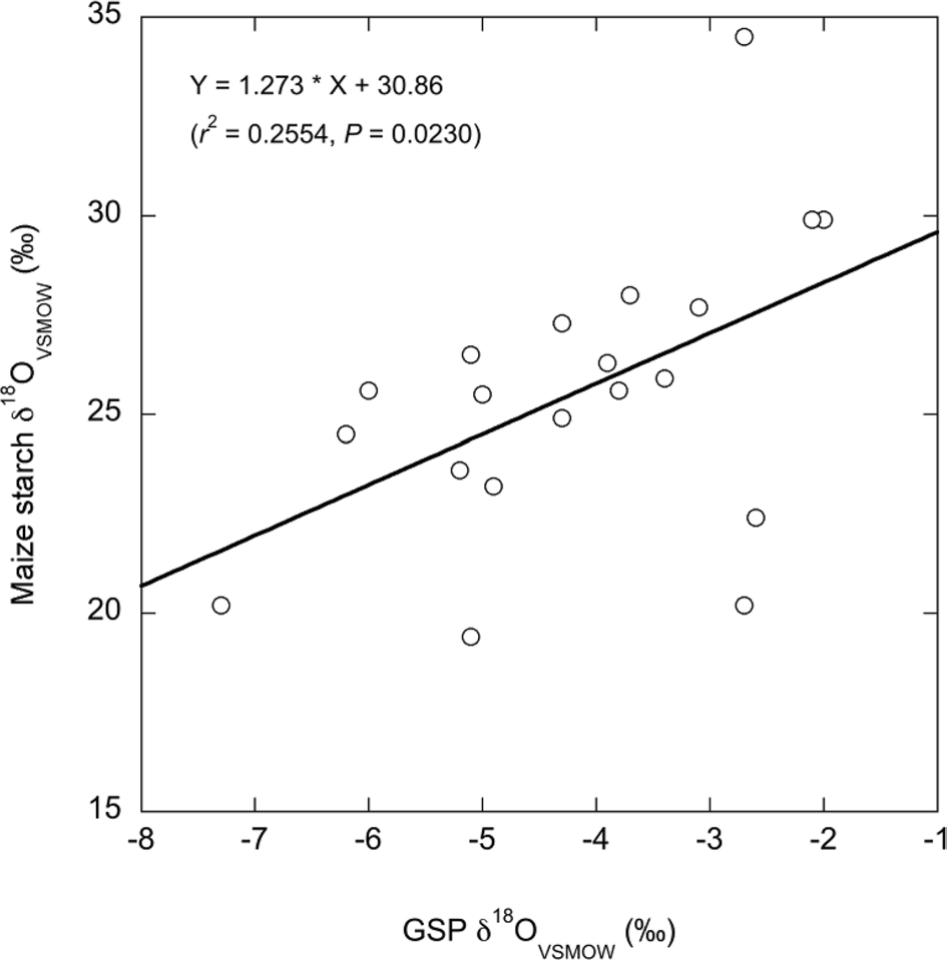
Plot of the sampled maize starch *δ*^18^O value versus the GSP *δ*^18^O value with linear regression line and equation.

*Values not used in linear regression model (see Fig. 1).

*DRC* Democratic Republic of the Congo.

### Isotope analysis

The relative amounts of two stable (i.e., non-radioactive) isotopes in the elements carbon (C), nitrogen (N), or oxygen (O) are presented as the ratio (R) of the heavy to light isotope—i.e., R = ^13^C/^12^C, ^15^N/^14^N, ^18^O/^16^O. Since these ratios are small, it is typical to express a sample ratio (R_samp_) in “delta notation” (δ) as parts per thousand (‰) difference relative to an internationally accepted standard zero-point (R_RM_), where δ = (R_samp_/R_RM_ − 1)^12^. The standard used for expressing a δ value varies, with C referenced to Vienna-Pee Dee Belemnite (VPDB), N referenced to the average N_2_ in the atmosphere (Air), and O referenced to Vienna Standard Mean Ocean Water (VSMOW)^13^.

Dry material was weighed into capsules that were crimped closed and then stored in covered 96-well PCR plates. Approximately 500 μg (± 10%) of sample material was sealed into tin capsules for C and N isotope ratio analysis. Approximately 100 μg (± 10%) of sample material was sealed into silver capsules for O isotope ratio analysis. Capsules prepared for O isotope ratio analysis were stored under vacuum until analyzed except benzoic acid reference materials as they would sublime. Laboratory reference materials for normalization of measured isotope ratios to the isotope δ scales and for quality control purposes were weighed at the same time as samples and included in each analytical sequence.

#### Carbon and nitrogen

Measurements of δ^13^C values, δ^15^N values, wt% C, and wt% N were performed using a Thermo Scientific MAT 253 isotope ratio mass spectrometer with an attached Costech elemental analyzer (ECS4010). Two laboratory reference materials of known δ^13^C_VPDB_ and δ^15^N_Air_ values, glutamic acids UU-CN-1 and UU-CN-2, were included at defined positions within the analytical sequence for correction of drift (time) and linearity (peak area), as needed, and for data normalization. UU-CN-1 had a calibrated δ^13^C value of + 23.328 ± 0.088‰ and δ^15^N value of + 49.28 ± 0.27‰. UU-CN-2 had a calibrated δ^13^C value of − 28.254 ± 0.039‰ and δ^15^N value of − 4.58 ± 0.01‰. A third laboratory reference material with long-term measured δ^13^C and δ^15^N values, glutamic acid UU-CN-3, was analyzed to provide a quality control assessment; this reference material has a long-term δ^13^C mean of − 12.629‰, within-sequence repeatability of 0.087‰, and between-sequence uncertainty of 0.039‰. It has a long-term δ^15^N mean of + 9.18‰, with within-sequence repeatability of 0.28‰, and between-sequence uncertainty of 0.12‰. Additionally, two commercially available starches (C3str1, a C_3_ plant starch, Aldrich Chemical Corp) and C4str3, a C_4_ plant starch, Sigma Chemical Co.) were analyzed alongside starch fractions for comparison of element concentrations.

#### Oxygen

Measurements of δ^18^O values were performed using a Thermo Scientific MAT 253 with an attached high temperature conversion elemental analyzer (TCEA). Laboratory reference materials of known δ^18^O values, benzoic acids UU-OH-5 and UU-OH-7, were included at defined positions within the analytical sequence for correction of drift (time) and linearity (area), as needed, and for data normalization. UU-OH-5 had a calibrated δ^18^O value of + 36.35 ± 0.18‰. UU-OH-7 had a calibrated δ^18^O value of − 2.78 ± 0.38‰. An additional laboratory reference material with long-term oxygen measurements, benzoic acid UU-OH-6, was analyzed to provide a quality control assessment. It has a long-term δ^18^O mean of + 26.06‰, within-sequence repeatability of 0.57‰, and between-sequence uncertainty of 0.17‰.

## Results

### Initial set of antimalarials

As an initial study of antimalarials, measured element concentrations and stable isotope ratios of 18 samples analyzed in bulk are presented in Table 1, grouped by API. Only 5 of the 18 samples contained nitrogen above the limit of quantitation of 1%. Note that not all APIs in the study contain detectable nitrogen (e.g., artesunate) so nitrogen cannot be a factor for predicting authenticity. Measured element concentrations ranged from 12 to 55% for C, 1.2–15.8% for N, and 21–50% for O. Measured stable isotope ratios ranged from − 27.8 to − 10.4‰ for δ^13^C values, + 2.3 to + 3.4‰ for δ^15^N values, and + 10.3 to + 30.9‰ for δ^18^O values.

Comparisons of genuine (*n* = 9) and falsified (*n* = 9) antimalarials, using Mann–Whitney tests, found that both wt% C and δ^13^C values were significantly different between the two quality classifications (wt% C: *p* = 0.0003; δ values: *p* < 0.0001). As compared to the genuine antimalarials, the falsified antimalarials had a significantly lower median wt% C (30 vs. 43%) and a higher median δ^13^C value (− 11.8 vs. − 25.5‰). At *p* < 0.05, there were no significant differences in either wt% O or δ^18^O values observed between the genuine and falsified samples. Nitrogen data were not statistically tested as the majority (13 of 18, 72%) of samples contained no measurable N; however, it should be noted that the only samples containing measurable N (*n* = 5) were genuine antimalarials.

The lower C concentrations observed for the falsified antimalarials suggest the addition of inorganic materials, such as minerals that have lower wt% C (0–12%) than most plant and animal products (40–55%). The higher δ^13^C values of the falsified antimalarials also suggest the addition of either mineral carbonates (e.g., chalk) or a C_4_-based organic material. Further examination of differences in element concentrations and stable isotope ratios between genuine and falsified antimalarials required a component-specific approach in which excipients are separated for analysis. A follow-on survey of 26 antimalarial samples that contained or claimed to contain artesunate or artemether-lumefantrine was used for this component-specific investigation.

### Follow-on set of artesunate/artemether antimalarials

The bulk C, N, and O element concentrations and stable isotope ratios of 26 additional samples containing (or claiming to contain) artesunate or co-formulated artemether-lumefantrine are presented in Table 2, grouped as genuine (*n* = 17) or falsified (*n* = 9). Like the observations made in the initial survey, only a few samples contained measurable N (*n* = 9). However, in contrast to the initial survey, three falsified antimalarials in the follow-on survey contained measurable N.

Measured element concentrations ranged from 29 to 49% for C, < 1.0–15.5% for N, and 20–49% for O. Measured stable isotope ratios ranged from − 27.7 to − 10.7‰ for δ^13^C values, − 3.1 to + 3.8‰ for δ^15^N values, and + 19.1 to + 28.0‰ for δ^18^O values.

Comparisons of the genuine and falsified antimalarials using Mann–Whitney tests found that both C concentrations and δ^13^C values were significantly different (%C: *p* < 0.0001; δ values: *p* < 0.0001). As compared to the genuine antimalarials, the median wt% C of the falsified antimalarials was significantly lower (37 vs. 43%) while the median δ^13^C value of the falsified antimalarials was significantly higher (− 12.5 vs. − 25.5‰). These results mirrored those from the initial survey, as did that there was no significant difference in either wt% O or δ^18^O values observed between the genuine and falsified antimalarials in the follow-on survey. Nitrogen data were not statistically tested as 17 of 26 (65%) of samples in the follow-on survey contained no measurable N; however, it should be noted that none of the falsified pills had greater than 1.1% N (w/w). As nitrogen content is not a useful metric for predicting authenticity for this general class of antimalarials, neither can the nitrogen isotope ratio be used in this manner.

### Both studies combined

A diversity of APIs and brands were included in both genuine and falsified categories in the initial and follow-on surveys (Supplementary Material-Tables 1 and 2). Between the two, 7 genuine Guilin Pharmaceuticals Co. artesunate samples and 9 falsified versions of this product were tested. Comparisons of the genuine versus falsified Guilin samples found that both δ^13^C and δ^18^O values were significantly different between the two quality classifications (Mann–Whitney; *p* = 0.0002 and 0.0288, respectively). For carbon, the median δ value of the falsified antimalarials was higher than the genuine antimalarials (− 10.8 vs. − 19.5‰). The mean δ^13^C value for genuine antimalarials was − 23.6‰ with a standard deviation (SD) of 3‰; for falsified antimalarials, the mean δ^13^C value was − 11.8‰ with an SD of 1‰. For oxygen, the opposite was found; the median δ value of the falsified antimalarials was lower than their genuine counterparts (+ 23.8 vs. + 27.6‰). The mean δ^18^O value for genuine was + 24.5‰ with SD = 3‰; for falsified, the mean δ^18^O value was + 24.3‰ with SD = 0.5‰.

As noted above, the higher δ^13^C values of the falsified antimalarials analyzed as bulk suggests the addition of C_4_ plant-based organic material—e.g., starch derived from maize^40^. Another possibility is the addition of inorganic carbonates, especially where the wt% C is low. To investigate the potential sources of the carbon-containing material present in the falsified antimalarials, the “starch” fraction was extracted from each sample and analyzed. We define the starch fraction as the material that was insoluble in a series of solvent washes (water, ethanol, acetone, and hexanes; see Methods).

The measured element concentrations of the starch fractions ranged from 30 to 41% for C and 32–51% for O (see Table 2). Considering just the falsified antimalarials in the follow-on survey (*n* = 9), all had starch fractions with δ^13^C values indicative of C_4_ plants (see Table 2). To identify the potential growth locations of the C_4_ starch used in these falsified antimalarials, we collected maize from continental US, Africa and Asia and examined the correlation between the isotopic composition of growth water and maize starch.

### Starch source predictions

The coordinates of known growth locations of collected maize (see Methods) were used to estimate mean annual precipitation (MAP) δ^18^O values through the Online Isotopes in Precipitation Calculator (OIPC)^53^ (Table 3). Monthly data from the OIPC were also used to calculate an average growing season precipitation (GSP) δ^18^O value for each collection location (Supplementary Material-Table 3). Growing seasons in the USA were identified using an online planting date calculator^54^ and those in Asia and Africa using online crop calendars^55^.

There was no significant correlation between the δ^18^O values of MAP and maize starch or between the δ^18^O values of GSP and maize starch (Pearson correlation coefficient, *p* > 0.05 for both). For the maize grown in Utah, USA, the relationship between the estimated δ^18^O values of MAP and GSP and the measured δ^18^O value of the starch was particularly counterintuitive. These water δ^18^O values were the lowest estimated from the OPIC, while the starch δ^18^O value was among the highest measured. Removing this sample from consideration, the correlation between the δ^18^O values of MAP and corn starch was still not significant; however, there was a significant correlation between the δ^18^O values of GSP and maize starch (*r* = 0.505, *p* = 0.023). The GSP average was not year-specific and may explain some of the unexplained residuals in the model.

We performed a linear regression with the dependent variable as the maize starch δ^18^O value and the independent variable as the GSP δ^18^O value. The slope was 1.273 ± 0.5123‰ (standard error) and the intercept was 30.86 ± 2.254‰ (Fig. 1). Uncertainties in the GSP as well as the measured δ^18^O values of the starch were considered insignificant relative to the residuals in the model; therefore, they were ignored. Using the inverse model, we calculated the δ^18^O values of growth water available to maize used as an excipient in the falsified antimalarials analyzed in the follow-on survey (see Table 4).

**Table 4:** Predicted GSP *δ*^18^O (‰) for the follow-on set (Table 2) using the inverse model for predicting maize starch from GSP.

ID Code	“Starch” *δ*^18^O (‰)	Predicted GSP *δ*^18^O (‰)	Standard error (‰)
42,005	26.4	− 4.0	0.3
Lao 05/22	31.1	− 3.1	0.5
G020	29.7	− 3.3	0.4
G021	31.9	− 2.9	0.6
G049	29.0	− 3.5	0.4
G054	28.9	− 3.5	0.4
G069	29.8	− 3.3	0.4
G215	26.4	− 4.0	0.3
G227	26.6	− 4.0	0.3
G244	32.9	− 2.7	0.7
G260	25.2	− 4.2	0.3
G261	28.4	− 3.6	0.4
G264	30.0	− 3.3	0.5
G265	25.7	− 4.1	0.3
G053	19.8	− 5.3	0.5
G050	25.1	− 4.3	0.3
G052	26.0	− 4.1	0.3
G015	26.0	− 4.1	0.3
Lao 07/24	27.9	− 3.7	0.3
Lao 07/25	28.3	− 3.6	0.4
Tan 09/02	30.6	− 3.2	0.5
Kenya 07/01	26.3	− 4.0	0.3
TAN 09/02	30.8	− 3.1	0.5
BEN 08/04	27.2	− 3.8	0.3
13011/1	23.7	− 4.5	0.3
12071	23.1	− 4.7	0.3
12063	23.2	− 4.6	0.3
CAM S5 2/08	28.8	− 3.5	0.4
CAM S5 3/08	28.7	− 3.5	0.4
CAM S5 4/08	28.6	− 3.6	0.4
CAM S5 5/08	28.7	− 3.5	0.4
Kenya 07/02	25.3	− 4.2	0.3

Notably, the stable isotope examination of the starch from falsified samples of artesunate labeled as “Guilin Pharmaceutical Co., Ltd” and “Mekophar Chemical Pharmaceutical Joint-Stock Co., Vietnam” (4 and 5 samples, respectively) both were derived from C4 plants, but the δ^18^O values in extracted starch differed sufficiently (23.6 ± 0.6 and 28.7 ± 0.1‰, respectively) to indicate that the starch was derived from different sources for the two falsified sample sets. This finding suggests different localities and/or different manufacturers of the two falsified sample sets with different labels.

## Discussion

The variation observed in the element concentrations and stable isotope ratios of antimalarials analyzed in bulk or as the extracted starch fraction suggests that IRMS may be a useful tool for profiling falsified antimalarials. Here, we were able to identify excipients as C_3_ or C_4_ plant-based materials based on measured δ^13^C values and found that C_4_ ingredients were exclusively used in falsified antimalarials as opposed to genuine antimalarials. By extracting and analyzing starch from maize grown in known locations, we may be able to predict potential growth water δ^18^O values for the starch present in falsified antimalarials. To improve predictions, future work would need to refine starch extraction methods, add more known-origin plants to the comparison database, collect more accurate data on growth water δ^18^O values, and generate specific prediction models for different starches commonly used as excipients in antimalarials.

Limitations of this pilot work include the relatively small sample size that precluded more detailed analysis and the lack of comprehensive information on the origins of the starch in both genuine and falsified samples to support the precipitation water isotope model. Further work is needed using the creation of simulated medicines with different API and excipient combinations, linked to isoscapes with a wider geographical spread of maize IRMS data, and the potential effect of technological processing on manufacturing food grade and pharmaceutical grade starch, to understand the potential accuracy of this technique in tracking the origin of falsified pharmaceuticals. A large overlap of regions with similar GSP δ^18^O values coupled with similar maize growing seasons limits the use of a simple oxygen isotope-based model to predict the location of maize-based excipients in falsified malarial drugs.

Established research has shown that stable isotope analysis can be used as a tool to elucidate the origin of many pharmaceutical products, both commercially and clandestinely made. More research is needed on a diversity of approaches for estimating the origins of pharmaceutical excipients and APIs, such as for water residues in tablets and liquids in falsified vaccines, using larger numbers of samples of known origin and greater diversities of pharmaceuticals (https://www.cghr.ox.ac.uk/research/medicine-quality-research-group/mqrg-projects/foresfa). If it is demonstrated to be helpful in providing actionable evidence for estimating falsified medicine origin, an international infrastructure for consensus protocols and appropriate data sharing will be needed.

### Supplementary Information

Supplementary Information.

## Data Availability

The datasets generated and analysed during the current study are available in Tables 1–3 and in the Supplementary Material-Tables 1–3.
